# Protective CD8+ T Cell Response Induced by Modified Vaccinia Virus Ankara Delivering Ebola Virus Nucleoprotein

**DOI:** 10.3390/vaccines10040533

**Published:** 2022-03-29

**Authors:** Alexandra Kupke, Asisa Volz, Erik Dietzel, Astrid Freudenstein, Jörg Schmidt, Hosam Shams-Eldin, Sylvia Jany, Lucie Sauerhering, Verena Krähling, Michelle Gellhorn Serra, Christiane Herden, Markus Eickmann, Stephan Becker, Gerd Sutter

**Affiliations:** 1 Institute of Virology, Philipps University Marburg, 35043 Marburg, Germany; kupke@staff.uni-marburg.de (A.K.); dietzel.erik@gmx.de (E.D.); schmidt3@staff.uni-marburg.de (J.S.); shamseld@staff.uni-marburg.de (H.S.-E.); sauerhel@staff.uni-marburg.de (L.S.); kraehliv@staff.uni-marburg.de (V.K.); michelle.gellhornserra@uni-marburg.de (M.G.S.); eickmann@staff.uni-marburg.de (M.E.); 2 German Center for Infection Research, Partner Site Giessen-Marburg-Langen, 35043 Marburg, Germany; 3 Institute of Virology, University of Veterinary Medicine Hannover, 30559 Hannover, Germany; asisa.volz@tiho-hannover.de; 4 German Center for Infection Research, Partner Site Munich, 80539 Munich, Germany; sutter@viro.vetmed.uni-muenchen.de; 5 Division of Virology, Institute for Infectious Diseases and Zoonoses, LMU Munich, 80539 Munich, Germany; astrid.freudenstein@viro.vetmed.uni-muenchen.de (A.F.); sylvia.jany@micro.vetmed.uni-muenchen.de (S.J.); 6 Institute of Veterinary Pathology, Justus Liebig University Giessen, 35392 Giessen, Germany; christiane.herden@vetmed.uni-giessen.de

**Keywords:** Ebola virus, Modified Vaccinia virus Ankara, vaccine, correlates of protection, nucleoprotein, glycoprotein

## Abstract

The urgent need for vaccines against Ebola virus (EBOV) was underscored by the large outbreak in West Africa (2014–2016). Since then, several promising vaccine candidates have been tested in pre-clinical and clinical studies. As a result, two vaccines were approved for human use in 2019/2020, of which one includes a heterologous adenovirus/Modified Vaccinia virus Ankara (MVA) prime-boost regimen. Here, we tested new vaccine candidates based on the recombinant MVA vector, encoding the EBOV nucleoprotein (MVA-EBOV-NP) or glycoprotein (MVA-EBOV-GP) for their efficacy after homologous prime-boost immunization in mice. Our aim was to investigate the role of each antigen in terms of efficacy and correlates of protection. Sera of mice vaccinated with MVA-EBOV-GP were virus-neutralizing and MVA-EBOV-NP immunization readily elicited interferon-γ-producing NP-specific CD8+ T cells. While mock-vaccinated mice succumbed to EBOV infection, all vaccinated mice survived and showed drastically decreased viral loads in sera and organs. In addition, MVA-EBOV-NP vaccinated mice became susceptible to lethal EBOV infection after depletion of CD8+ T cells prior to challenge. This study highlights the potential of MVA-based vaccines to elicit humoral immune responses as well as a strong and protective CD8+ T cell response and contributes to understanding the possible underlying mechanisms.

## 1. Introduction

The Ebola virus (EBOV) belongs to the family of *Filoviridae* within the order *Mononegavirales* and is one of the causative agents of the Ebola virus disease (EVD), which can lead to gastrointestinal disorders such as vomiting and diarrhea, disseminated intravascular coagulation and multi-organ failure with 30–90% of cases being lethal [1,2]. Fruit bat species have been discussed as a reservoir of this zoonotic disease [3,4] but also other wildlife species, including great apes, can serve as a source of infection during hunting and butchering [5,6,7]. Human-to-human transmission is mainly mediated by direct contact with infectious body fluids [1,8].

EBOV outbreaks have been recorded in endemic areas in Africa repeatedly since 1976 with usually only a limited number of people affected in small, mostly rural areas [2]. However, the large EBOV outbreak in West Africa (2014–2016) with Guinea, Sierra Leone and Liberia carrying the highest burden of disease, has shown that the virus can efficiently and rapidly spread from small endemic areas to large cities and non-endemic countries. The outbreak was therefore declared as a Public Health Emergency of International Concern by the WHO [9]. With more than 28,600 people infected and 11,300 fatalities, this outbreak has revealed substantial deficits concerning the development of vaccines and antiviral therapeutics against highly pathogenic viruses. Though decades of virus research have led to several promising anti-EBOV vaccine candidates [10], it was not before the beginning of the West African outbreak that safety and tolerability trials in humans were performed. During the 2014–2016 outbreak in West Africa and several outbreaks in the Democratic Republic of Congo 2018–2020, the development of the most promising vaccine candidates, amongst them the live-attenuated recombinant vesicular stomatitis virus-based vaccine (rVSV-ZEBOV [11,12,13]) and the combination of an adenovirus serotype 26 (Ad26.ZEBOV-GP) and the Modified Vaccinia virus Ankara (MVA)-based vaccine MVA-BN-Filo [14,15], was accelerated.

The rVSV-ZEBOV vaccine candidate, which targets the EBOV glycoprotein (GP), proved to be highly efficacious (efficacy rate of up to 100% [16]). However, during the clinical phase I and II trials, side-effects occurred in a dose-dependent manner, especially in one of the cohorts [12,13]. These side-effects underline that the rVSV-ZEBOV vaccine is a highly valuable vaccine candidate in emergency scenarios when more severe reversible adverse effects might be assessed tolerably e.g., in contrast to planned vaccination of medical staff in endemic areas. Efficacy data for the Ad26/MVA-based vaccine, which targets four different filoviral proteins, is not yet available. Nevertheless, safety and immunogenicity studies have been performed successfully in humans [17,18,19]. However, a prime-boost vaccination with two different vaccines might be difficult for organizational reasons and production processes, especially in an outbreak scenario. Prime only or homologous prime-boost schedules may minimize costs and organizational efforts, especially in countries where shipment to rural areas is necessary.

The MVA vector we used in the present study in a homologous prime-boost schedule can serve as an efficient and safe vector vaccine platform [20,21]. Developed through 570 passages in chicken embryo fibroblasts (CEF), MVA has lost its ability to productively replicate in cells of human origin [22,23,24] which is an important safety feature of MVA and is strongly supported by in vivo distribution studies in non-human primates [25]. Furthermore, an MVA clinical candidate vaccine producing the spike glycoprotein of Middle East Respiratory Syndrome coronavirus (MERS-CoV) remained only transiently associated with tissues of the intramuscular inoculation site and the draining lymph nodes, suggesting continuous clearance of the MVA vaccine [26]. Moreover, safety of recombinant MVA has been shown in multiple clinical studies [27,28]. Clinical applications in HIV-positive individuals demonstrated the innocuous use of recombinant and non-recombinant MVA vaccines in immunocompromised individuals [29,30]. Despite its replication-deficiency in human cells, MVA is highly immunogenic and serves as an efficient vaccine with essentially all vector virus particles of a vaccine preparation expressing the recombinant target gene products. Moreover, MVA encompasses superb immune stimulating properties [31,32,33,34,35,36]. Recently, a recombinant MVA-based vaccine capable of producing Ebola virus-like particles induced EBOV-neutralizing antibodies and was shown to be effective after a single vaccination in a nonhuman primate model, demonstrating the capacity of this vector platform in EBOV-specific vaccine development [37].

Non-human primates (NHPs) are the animal model of choice to study the clinical and immunological aspects of EVD [38]. However, due to limited access to BSL4 labs with NHPs facilities and because of ethical concerns, rodent models may be a good choice to study certain aspects, e.g., the immune response of vaccines in terms of neutralizing antibodies and virus-specific T cell responses. Because wildtype mice are not susceptible to non-rodent-adapted EBOV we decided to use the type I interferon-deficient mouse model (IFNAR^-/-^) [39] which enables the use of a wildtype EBOV for infection.

Until now, the mechanisms which lead to a protective immune response against EBOV have not been completely understood. Virus-neutralizing antibodies directed against the only EBOV surface protein GP seem to correlate with protective immunity. Thus, the EBOV GP is considered as the key immunogen and most EBOV candidate vaccines in development deliver GP alone or together with other viral antigens [40,41,42,43]. Moreover, previous studies in animal models demonstrated the role of EBOV antigen-specific CD8+ T cell responses for preventing fatal EBOV disease after challenge [37,40,41,42,43,44,45,46].

In this study, we investigated the immunogenicity and protective capacity of the EBOV nucleoprotein (NP) when delivered as privileged antigen by a candidate clinical MVA vector vaccine. NP is not present on the surface of Ebola virions nor is it predicted to be expressed on the membrane of MVA-infected cells [47,48,49]. Thus, NP-specific protective immunity is more likely based on CD8+ T cell responses, and antiviral cytotoxic CD8+ T cell immunity is a key feature of rapidly protective MVA vaccination [50,51]. We generated two MVA-based candidate vaccines expressing EBOV NP (MVA-EBOV-NP) or the surface antigen GP (MVA-EBOV-GP) to investigate the role of the respective antigen in terms of correlates of protection after homologous prime-boost immunization. MVA-EBOV-NP vaccination in an IFNAR^-/-^ mouse/EBOV challenge model resulted in the induction of solid protective immunity at levels similar to those obtained with MVA-EBOV-GP immunization. While MVA-EBOV-NP failed to elicit significant amounts of EBOV neutralizing antibodies, it induced high levels of interferon gamma producing CD8+ T cells as demonstrated by ELISPOT for two different NP epitope specificities. Importantly, antibody-mediated depletion of CD8+ T cells abolished the protection of MVA-EBOV-NP immunized animals upon EBOV challenge, suggesting NP-specific cytotoxic T cells as correlates for candidate vaccine efficacy. This study highlights the potency of MVA-based vaccines to elicit a strong and protective CD8+ T cell response. As witnessed during the SARS-CoV-2 pandemic, neutralizing antibody responses are prone to being affected by antigen mutations while T cell epitopes remain largely unimpaired [52] and therefore, focusing on protective CD8+ T cell responses might be crucial for future vaccine development.

## 2. Materials and Methods

### 2.1. Plasmid Construction

We modified the cDNA sequences coding for the nucleoprotein (NP) and the glycoprotein (GP) of EBOV by introducing silent codon alterations to remove runs of guanines or cytosines which may prevent frameshift mutations during vaccinia virus DNA replication and assure the genetic stability of the recombinant MVA genome. In addition, we inactivated three signal sequences (TTTTTNT) to prevent a premature termination of vaccinia virus-specific early transcription. The optimized GP and NP gene sequences were generated by DNA synthesis (GENEWIZ, LLC., South Plainfield, NJ, USA) and inserted into the MVA vector plasmids pIIIH5red and pLW-73, respectively, to obtain the MVA expression plasmids pIIIH5red-EBOV-GP and pLW-73-EBOV-NP (Figure S1) [53,54]. In these plasmids both recombinant genes were placed under the transcriptional control of the synthetic vaccinia virus early/late promoter PmH5 [55].

### 2.2. Generation of Recombinant MVA Viruses

The generation of the recombinant MVA-EBOV viruses was essentially performed as described previously [54]. Briefly, the MVA clonal isolate F6 [22,27] was used as virus starting material and propagated on primary chicken embryo fibroblasts (CEF) obtained from 10-day old embryonated SPF chicken eggs (Valo BioMedia GmbH, Osterholz-Scharmbeck, Germany). MVA-EBOV-NP and MVA-EBOV-GP were obtained following transfection of MVA-infected CEF with vector plasmid DNA and clonally isolated in plaque passages on CEF monitoring for the transient co-expression of the fluorescent marker proteins GFP or mCherry.

Recombinant MVA primary stock viruses were grown in CEF and served for further analysis in quality control experiments. Genetic identity and genetic stability of the vector viruses were assessed by PCR analysis of genomic viral DNA. Replicative capacities of the recombinant MVA were tested in one-step and multiple-step growth experiments in CEF using virus titration in plaque forming units (PFU). Similarly, the replication deficiency of both recombinant viruses in cells of human origin was confirmed by growth analysis in HaCat cells [56]. To generate vaccine preparations, the recombinant MVA-EBOV viruses were amplified in CEF, purified by ultracentrifugation through sucrose, plaque-titrated in CEF, reconstituted to vaccine stocks in Tris-buffered saline pH 7.4 and stored at −80 °C until usage.

### 2.3. Characterization of Recombinant MVA Genomes

Genetic identity and genetic stability of vector viruses was confirmed by polymerase chain reaction (PCR) using genomic viral DNA as described previously [54]. Briefly, genetic identity and integrity of recombinant MVA was confirmed by PCR monitoring for the characteristic six major deletions in the MVA genome. MVA-DNA is analyzed by six different PCR reactions using oligonucleotide primers that are designed to amplify highly specific DNA fragments extending over the six major deletions sites within the MVA genome.

The genetic stability of the recombinant MVA was monitored using two different PCR assays demonstrating the site-specific insertion of the heterologous EBOV gene sequences in the MVA genome. The same insertion site-specific PCR reactions (deletion III site, intergenic site 069R-070L) were used to confirm the proper removal of the marker genes encoding the fluorescent proteins mCherry and GFP from the genome of final recombinant viruses. The precise intragenomic deletion of the mCherry marker gene was revealed by amplification of a 2.921 kb DNA fragment corresponding to the expected molecular weight of the PmH5-EBOV-GP gene expression cassette inserted at the site of deletion III. The deletion III site-specific control PCR reaction amplified the characteristic 0.762 kb DNA fragment from genomic, non-recombinant MVA DNA. Similarly, the intragenomic deletion of the GFP marker gene was demonstrated by amplification of a 2.653 kb PCR product corresponding to the expected molecular weight of the PmH5-EBOV-NP gene expression cassette inserted at the intergenic site 069R-070L of the MVA genome. The intergenic site 069R-070L-specific control PCR amplified a characteristic 0.457 kb DNA fragment from genomic, non-recombinant MVA DNA.

### 2.4. Detection of Recombinant EBOV Proteins

CEF were infected with recombinant MVA-EBOV viruses at the multiplicity of infection (MOI) of 5. Non-recombinant MVA (MVA) or mock-infected cells served as controls. Total cell extracts were prepared at 8, 16, 24 and 48 h post infection (hpi). After 10% SDS-PAGE proteins were analyzed by western blots using anti-EBOV GP (1G12); [57] or anti-EBOV NP antibodies [58] as primary antibodies. Goat anti-mouse HRP-conjugated antibody (Sigma, St. Louis, MO, USA; 1:5000 dilution) and goat anti-chicken HRP-conjugated antibody (Thermo Fisher, Waltham, MA, USA; 1:10,000 dilution) served as secondary antibodies for detection of EBOV GP and EBOV NP using MicroChemisystems (biostep, Burkhardtsdorf, Germany).

### 2.5. Immunization and EBOV Infection in Mice

Male and female type I interferon receptor-deficient (IFNAR^-/-^) mice [59] were used as an infection model since wildtype mice are not susceptible to EBOV infection [60]. They have been 20-fold backcrossed to the C57BL/6 background and were kept under specified pathogen-free conditions at the animal facilities of TWINCORE, Hannover, Germany and LMU Munich, Germany. Six- to ten-week-old mice were immunized two times intramuscularly in the quadriceps muscle with 10^8^ PFU of either MVA-EBOV-NP or MVA-EBOV-GP using a prime/boost regimen at a 21-day interval. Serum samples were obtained at days 0, 18 and 31 after the first vaccination at the facial vein. EBOV infection was performed according to an adapted protocol [39] at the animal facility of the high safety laboratory (BSL4) of the Philipps University Marburg, Germany, where mice were anesthetized with a short isoflurane anesthesia and received 1000 PFU of the wildtype Mayinga isolate of EBOV Zaire diluted in 30 µL sterile, pre-warmed DMEM intranasally. Intranasal infection was performed to mimic infection of mucous membranes. Mice were kept in groups of a maximum of five individuals in isocages (Tecniplast, Hohenpeißenberg, Germany) and were examined daily for body weight, general condition and spontaneous behavior over a maximum of 14 days after infection. Parameters measured resulted in a clinical score and the clinical end point was defined as a score of 10 or of 6 on two consecutive days (Table S1). Serum samples were taken at 5, 9 (data not shown) and 14 days post infection (dpi).

### 2.6. Analysis of Antibody Response

Serum samples were analyzed by whole-virion ELISA according to a previously published protocol [61]. Briefly, microtiter plates coated with EBOV (Zaire) antigen were washed three times with PBS + 0.1% Tween^®^20 (PBST) and blocked for 45 min with PBS containing 5% milk powder. Mouse sera were diluted 1:200 in PBST containing 1% milk powder and incubated on the microtiter plates for 1 h. Polyclonal HRP-coupled antibodies (DAKO, Santa Clara, CA, USA), diluted 1:1000 and incubated for 30 min, were used for detection. One hundred microliters of 3,3′,5,5′-tetramethylbenzidine (TMB) substrate solution (SureBlueTM TMB Microwell Peroxidase Substrate, KPL Inc., Gaithersburg, MD, USA) was incubated in each well for 10 min protected from light. The reaction was stopped with 100 μL/well of TMB-Stop Solution (KPL Inc., Gaithersburg, MD, USA), and the optical density (OD) was determined at 450–630 nm using an automated spectrophotometer (PHOmo, Autobio Labtec Instruments Co., Ltd., Zhengzhou, China) within 5 min. Each sample was analyzed in duplicates, and the mean OD value of each sample on mock antigen was subtracted from the OD value on Zaire EBOV antigen.

Further, sera were analyzed for EBOV-neutralizing antibodies by virus neutralization assay as described before [13]. In order to inactivate complement, mouse sera were incubated at 56 °C for 30 min. After centrifugation at 13,000 rpm for 10 min, duplicates of sera were serially diluted starting from 2^3^ to 2^10^ in DMEM supplemented with 2% fetal calf serum (FCS, Gibco), penicillin (100 U/mL), streptomycin (100 mg/mL) and L-glutamine (2 mmol/L) (all from Invitrogen, Waltham, MA, USA) in 96 well culture plates. A total of 100 TCID50 units of EBOV (Zaire, isolate Mayinga, AF086833) were added to the serum dilutions and incubated for 1 h at 37°C. Afterwards, Vero cell suspension in DMEM containing 2% FCS was added. Plates were then incubated at 37 °C with 5% CO_2_ and cytopathic effects (CPE) were evaluated at seven days post infection. Neutralization titers were calculated as geometric mean titers.

### 2.7. Analysis of T Cell Response

Mice were sacrificed eight days post prime or prime-boost immunizations. A cell suspension was prepared by homogenizing the spleens through 200 µm mesh sieves and red blood cells were removed by adding Red Cell lysis buffer (Sigma, St. Louis, MO, USA). After centrifugation, the cell pellet was resolved in RPMI medium supplemented with 10% FCS, 2 mM L-glutamine and 100 IU/mL penicillin/streptomycin. Interferon-γ secreting CD8+ T cells were analyzed by ELISPOT assay (ELISPOTPLUS Kit for mouse IFN-γ, MABTECH, Eching, Germany) following the manufacturer’s instructions. ELISPOT plates were pre-incubated overnight with the antibody solution and then incubated with the cell suspension that had been stimulated with the EBOV-specific peptides NP_44–52_ (YQVNNLEEI; [45]) and NP_388–396_ (FQQTNAMVT; [45]). The spots were counted and analyzed by using an automated ELISPOT plate reader and software following the manufacturer’s instructions (A.EL.VIS Eli.Scan, A.EL.VIS ELISPOT Analysis Software, Hannover, Germany).

### 2.8. Determination of EBOV Loads and Infectious Virus in Mouse Organs and Sera

Samples of immunized and challenged mice were excised from lung, liver and spleen and homogenized in 1 mL DMEM with ceramic and glass beads (Lysing Matrix H 2 mL tubes, MP Biomedicals, Illkirch-Graffenstaden, France) in a Mixer Mill MM 400 (Retsch, Haan, Germany) instrument three times for 5 min. Homogenates were centrifuged for 5 min at 2400 rpm in a Mikro 200R centrifuge (Hettich Lab Technology, Tuttlingen, Germany) to remove tissue debris. Aliquots of 100 µL of supernatants or 10 µL of mouse sera were used for RNA isolation with either the RNeasy Mini Kit (Qiagen, Hilden, Germany) or the QIAamp Viral RNA Mini Kit (Qiagen, Hilden, Germany) according to the manufacturer’s instructions. The RNA amount of organ homogenates was measured by using the NanoDrop ND-100 spectrophotometer. Total RNA was reverse transcribed and quantified by the means of a standard curve based on a real time RT-PCR protocol which has been previously published to differentiate between EBOV virus subtypes Sudan and Zaire [62] and adapted to our lab. Briefly, the One Step RT Kit (Qiagen, Hilden, Germany) was used in combination with the primer pair (forward TGGGCTGAAAAYTGCTACAATC, reverse CTTTGTGMACATASCGGCAC) and probes (6FAM-TTACCCCCACCGCCGGATG-BHQ1, 6FAM-CTACCAGCAGCGCCAGACGG-BHQ1) on an ABI StepOnePlus Real-Time PCR System (Life Technologies Instruments, Waltham, MA, USA). Cycling steps were as follows: 30 min 50 °C, 15 min 95 °C, followed by 45 cycles 95 °C for 15 s and 58 °C for 30 s.

Live virus titers in mouse sera obtained at the clinical end point were determined by immunoplaque assay on Vero cells. In short, sera were diluted in a 10-fold series in DMEM and incubated on Vero E6 cells for 1 h at 37 °C. After removal of infectious medium cells were covered with 2% CMC (carboxymethylcellulose)/MEM. Cells were fixed 6 dpi and stained for EBOV proteins with a polyclonal goat serum against EBOV (clone 36) and a donkey anti-goat antibody labeled with Alexa Fluor 488 (Life Technologies, Waltham, MA, USA) as secondary antibody. Subsequently, plaques per well were counted and titers were calculated per ml serum.

### 2.9. Clinical Serum Chemistry

To determine levels of liver enzymes aspartate and alanine aminotransferase (AST and ALT, respectively), as a sign for hepatocellular damage, serum clinical chemistry was assessed with the Piccolo Xpress Chemistry Analyzer and the General Chemistry 13 panel (both Abaxis, Union City, CA, USA) according to the manufacturer’s instructions.

### 2.10. Histopathological Examination and In Situ Hybridization of Mouse Organs

Tissue samples of lung, liver and spleen were collected at the clinical end point or 14 dpi when the trial was ended and fixed in 10% neutral buffered stabilized formalin for 7 days under several changes of the fixative. Small pieces were routinely embedded in paraffin and sections were cut with a Leica RM2255 microtome (Leica Biosystems, Wetzlar, Germany) and stained with hematoxylin and eosin (H&E). For in situ hybridization, digoxigenin-labeled RNA probes which either targeted the viral genome (negative strand) or the corresponding mRNA (positive strand) coding for EBOV glycoprotein (GP) were constructed according to a protocol previously published [63,64]. Briefly, total viral RNA was isolated from virus stock (Mayinga strain; GenBank^®^ accession number NC_002549.1), reversely transcribed and amplified. A sequence of 1663 nt, coding for EBOV GP (gene ID 911829) was inserted in a pCR™4-TOPO^®^ TA vector using the TOPO TA Cloning^®^ Kit (Thermo Fisher, Waltham, MA, USA) and One Shot^®^ TOP10 Chemically Competent *E. coli*. Plasmid DNA was isolated with the E.Z.N.A.^®^ Plasmid DNA Mini Kit I and purified with HiBind^®^mini columns (both Omega Bio-tek, Norcross, GA, USA) according to manufacturer’s instructions. For detection of genomic RNA EBOV antisense primer (5959F; 5′-AGA GTA GGG GTC GTC AGG TC-3′, 20 nt, position 5959–5978) was combined with M13 forward primer (17 nt) and for detection of mRNA the EBOV GP sense primer (7621R; 5′-TCC GAT TGC AGC ACC TTC AT-3′, 20 nt, position: 7602–7621) was combined with an M13 reverse primer (17 nt). Inserts were amplified on an MWG Biotech Primus thermocycler (Ebersberg, Germany) with the following steps: initial denaturation at 94 °C for 3 min, 40 cycles comprising the following steps: 94 °C for 30 s, 50 °C for 30 s and 1 min at 72 °C. Afterwards, a final elongation for 10 min at 72 °C followed. PCR products were cleaned up to remove spare primers and nucleotides with the E.Z.N.A.^®^ Probe Purification Kit (Omega Bio-Tek, Norcross, GA, USA) according to the manufacturer’s recommendation. The DNA amount was determined with the NanoDrop^TM^ Lite Spectrophotometer (Thermo Fisher, Waltham, MA, USA). In vitro transcription was carried out with the HiScribe^TM^ T7 High Yield RNA Synthesis Kit (New England Biolabs, Frankfurt, Germany) for T3 RNA polymerase as well as DIG-UTPs (both Sigma, St. Louis, MO, USA) according to the protocol supplied and resulted in a probe length of 575 nt.

ISH was performed according to previously published protocols [65]. Briefly, 4 µm thick tissue sections were placed on glass slides (Superfrost Plus^®^, R. Langenbrinck, Emmendingen, Germany). Tissue was deparaffinized and rehydrated in a descending alcohol series. Afterwards, proteolytic digestion, post-fixation, acetylation and prehybridization followed. Hybridization of the probes was carried out overnight in a humid chamber at 69.5 °C. After several washing steps, non-bound RNA was removed by incubation with a mixture of RNases A and T (Roche diagnostics, Basel, Switzerland). As a detection system an anti-DIG antibody, labeled with alkaline phosphatase (Roche diagnostics, Basel, Switzerland), was used in combination with the substrates nitroblue tetrazolium chloride (NBT) and 5-bromo-4-chloro-3-indolyl phosphate (BCIP, both Sigma-Aldrich, St. Louis, MO, USA). Finally, slides were mounted with Kaisers Glyceringelatine (Merck, Darmstadt, Germany). Incubation of slides with hybridization reagents not containing the probes were used as negative controls.

### 2.11. Depletion of CD8+ T Cells and Flow Cytometric Analysis

To assess the role of the NP-specific CD8+ T cells in protective MVA vaccination, two groups of mice were vaccinated with the MVA-EBOV-NP vaccine candidate according to the scheme previously described (see Section 2.5) with one group being treated four times with an anti-CD8+ (clone 2.43) mouse monoclonal antibody purchased from Harlan Bioproducts, Indianapolis, USA. This T cell depletion was performed by administration of 100 µg anti-CD8+ antibody on days −2, 0, +2 and +4 prior to or after EBOV challenge on day 0. MVA-vaccinated mice were used as controls for clinical outcome. Infection was performed as described above and blood samples were gained at 4 dpi as well as at the clinical end point or at the end of the trial for the surviving individuals. In parallel, a group of uninfected, MVA-vaccinated mice were used to confirm successful depletion of immune cells by flow cytometric analysis of blood cells from antibody treated animals. Here, blood samples were taken every other day, except on day 6. Approximately 10^6^ cells were stained in 50 µL PBS supplemented with 3% FCS using monoclonal antibodies obtained from Biolegend (San Diego, CA, USA). T cells were detected using PE-labeled CD3+, PE-Cy7-labeled CD4+ and FITC-labeled CD8+ antibodies. To ensure specificity of staining, all staining tests contained negative controls from mice that had been mock-vaccinated/infected with PBS. Stained cells were analyzed with MACS Quant VYB and MACSQuantify™ Software (Miltenyi Biotec, Bergisch Gladbach, Germany).

### 2.12. Statistical Analysis

All statistical analyses were performed with GraphPad Prism Version 9 (GraphPad software). Mann–Whitney U test was used to calculate significances for virus-neutralizing titers, ELISPOT assays, for genome copies in organ homogenates and sera, for plaque-forming units determined by immunoplaque assay as well as serum levels of liver enzymes. Two-Way ANOVA and Tukey’s test for multiple comparisons were used to test if optical density values (ODs) measured by whole-virion ELISA were significantly different between time points. To determine if weight changes in mice were significantly different between groups, we used Two-Way ANOVA and Dunnett’s test for multiple comparisons. Differences in Kaplan–Meier survival curves were assessed by the use of Log-rank (Mantel–Cox) test.

## 3. Results

### 3.1. Construction and Characterization of Recombinant MVA Expressing EBOV NP or GP

**Figure 1:**
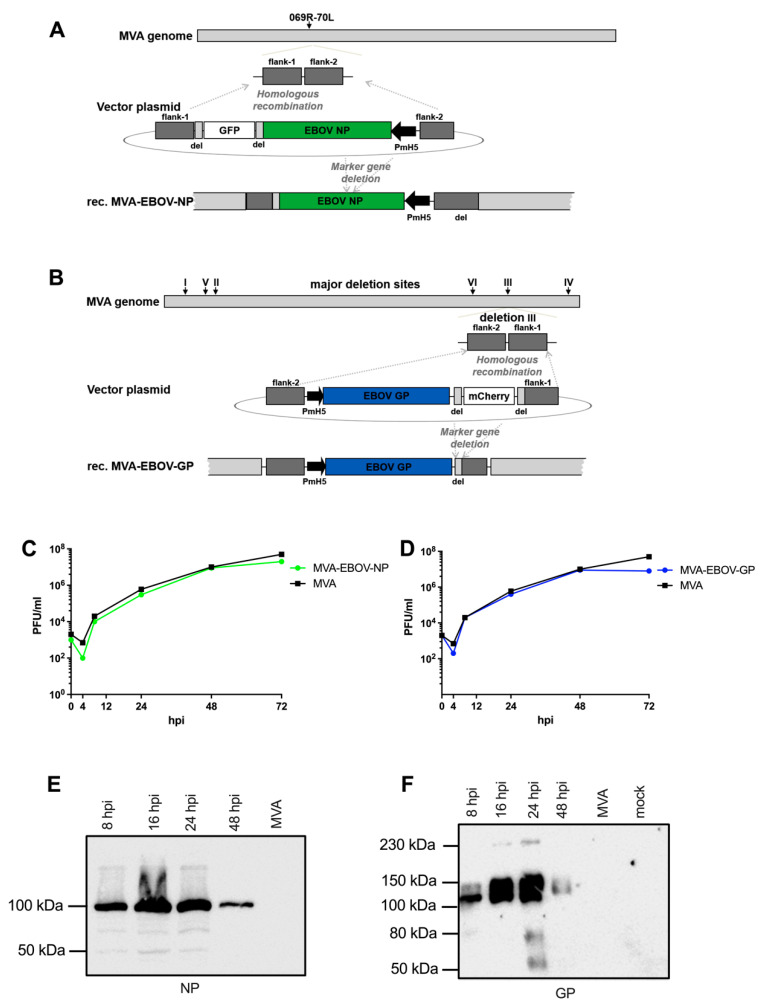
Generation of recombinant MVA-EBOV viruses. (**A**,**B**) Schemes of the MVA genome with the intergenic insertion site 069R-070L (**A**) or the major deletions sites I-VI (**B**). Flank-1 and flank-2 refer to MVA DNA sequences targeting the intergenic site 069R-070L (**A**) or deletion site III (**B**) in the MVA genome for insertion of recombinant genes. MVA vector plasmids contain recombinant EBOV NP or GP gene sequences, respectively, under transcriptional control of the vaccinia virus promoter PmH5 and a marker gene sequence for transient expression of the fluorescent protein GFP (**A**) or mCherry (**B**). Short repetitive sequences of MVA DNA (del) served to remove the marker genes by intragenomic homologous recombination (marker gene deletion). (**C**,**D**) Multiple-step growth analysis of recombinant MVA-EBOV viruses. Growth of recombinant viruses MVA-EBOV-NP (**C**) or MVA-EBOV-GP (**D**) was monitored upon infection of chicken fibroblast cells (**C**,**E**,**F**); hpi: hours post infection. (**E**,**F**) Synthesis of recombinant EBOV NP (**E**) and EBOV GP (**F**) was tested by Western blot analysis using cell lysates and supernatants from infected CEF cells. Polypeptides were separated by SDS–PAGE and tested by immunoblotting using either EBOV NP- or GP-specific antibodies. Uninfected cells (mock) or non-recombinant MVA-infected cells (MVA) served as controls.

The recombinant MVA-EBOV viruses (MVA-EBOV-NP and MVA-EBOV-GP) were formed by homologous recombination in MVA-infected chicken embryo fibroblasts (CEF) that were transfected with MVA vector plasmid DNA. The flanking MVA DNA sequences in these vector plasmids (Figure S1) precisely directed the incorporation of the EBOV recombinant gene sequences into the selected insertion sites of the MVA genome (as schematically depicted in Figure 1A,B). The expression cassette encoding EBOV NP was inserted into the intergenic site between the MVA open reading frames 070L and 069R genes (Figure 1A) [66] and the EBOV GP gene sequences into the deletion site III of the MVA genome (Figure 1B) [23,53].

Transient production of fluorescent marker proteins (green fluorescent protein GFP for MVA-EBOV-NP and red fluorescent mCherry for MVA-EBOV-GP) allowed to readily visualize infected cell foci and to clonally isolate recombinant viruses in plaque passages. The marker gene sequences are flanked by repetitive sequences of MVA DNA (del) to allow for precise marker gene deletion by intragenomic homologous recombination. The removal of the fluorescent marker genes by secondary intragenomic homologous recombinations resulted in the final marker free recombinant viruses MVA-EBOV-NP or MVA-EBOV-GP as confirmed by PCR analysis of the viral genomic DNA (data not shown).

Multiple-step-growth experiments on CEF served to analyze the growth behavior of the MVA-EBOV recombinant viruses (Figure 1C,D). In CEF, a cell substrate routinely used for industrial MVA vaccine production, the recombinant MVA-EBOV-NP and MVA-EBOV-GP propagated to titers similar to those obtained with non-recombinant MVA (MVA) and increased infectivities by approximately three to four orders of magnitude within 48 h of infection. The expression of the heterologous EBOV genes under transcriptional control of the strong synthetic early/late vaccinia virus specific promoter PmH5 resulted in the synthesis of readily detectable amounts of recombinant GP and NP proteins as shown by Western blot analysis of infected CEF cell lysates using EBOV NP- or GP-specific antibodies (Figure 1E,F). In lysates of CEF infected with MVA-EBOV-NP, we demonstrated the synthesis of an NP-specific protein with an appropriate molecular mass of about 100 kDa (Figure 1E); [67,68]. Easily noticeable amounts of NP were found in CEF lysates as early as 8 hpi. Recombinant NP protein production seemed to reach optimal levels within 24 hpi with lesser amounts of NP being visible at 48 hpi. Similarly, upon infection with MVA-EBOV-GP we specifically detected major protein bands corresponding to EBOV GP products in the estimated molecular masses of about 125–140 kDa (Figure 1F) [67,69,70]. The GP polypeptides were also revealed in the cell lysates as early as 8 hpi and remained detectable with increasing amounts until 24 hpi (Figure 1F). Again at 48 hpi the levels of apparent EBOV GP seemed to decline which is likely due to the cytopathic effect in the MVA infected CEF and the resultant release of recombinant protein to the medium supernatant of the cell cultures [69,71].

### 3.2. EBOV-Specific Antibodies Are Induced by MVA-EBOV-NP and -GP

**Figure 2:**
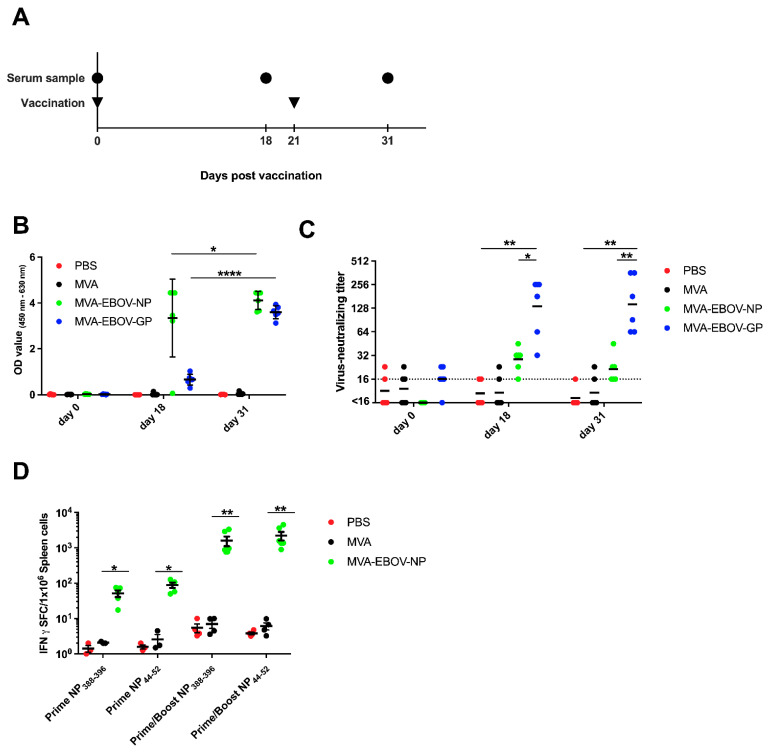
Immunogenicity of recombinant MVA-EBOV vaccines. (**A**) IFNAR^-/-^ mice were vaccinated intramuscularly with either PBS or 10^8^ PFU non-recombinant MVA (MVA) as controls or with 10^8^ PFU of the recombinant viruses MVA-EBOV-NP and MVA-EBOV-GP in a prime-boost regimen. Serum samples were taken at the indicated time points. (**B**) EBOV-virion ELISA performed with serum samples obtained at days 0, 18 and 31 after the prime vaccination of mock-vaccinated and vaccinated mice. Mean optical density values were measured at 450–620 nm; error bars: standard deviation; PBS: *n* = 5 mice, MVA, MVA-EBOV-GP, MVA-EBOV-NP: *n* = 6 mice. (**C**) Geometric mean EBOV-neutralizing titer determined by virus neutralization assay on days 0, 18 and 31 after prime vaccination, respectively; dotted line: limit of detection; PBS: *n* = 5 mice, MVA, MVA-EBOV-GP, MVA-EBOV-NP: *n* = 6 mice. (**D**) EBOV NP-specific CD8+ T cell response was measured by ELISPOT assay. Splenocytes were prepared at eight days after prime or prime/boost vaccination. NP_388–396_ or NP_44–52_ epitope-specific, IFN-spot forming CD8+ T cells (IFN-SFC) were quantified; bar: mean, error bars: standard error of the mean; PBS, MVA-prime NP: *n* = 3 mice; PBS, MVA-prime/boost NP: *n* = 4 mice; MVA-EBOV-NP-prime NP: *n* = 5 mice; MVA-EBOV-NP-prime/boost: *n* = 6 mice. * *p* < 0.05; ** *p* < 0.01, **** *p* < 0.0001.

To test the overall immunogenicity of the recombinant MVA-EBOV candidate vaccines, IFNAR^-/-^ mice were prime-boost vaccinated with MVA-EBOV-NP, MVA-EBOV-GP, non-recombinant MVA or PBS using a 21-day interval and serum samples were taken at days 0, 18 and 31 (Figure 2A). First, serum samples were analyzed for EBOV-specific antibodies using a whole-virion IgG ELISA (Figure 2B) [61]. As early as 18 days post vaccination (dpv), EBOV-specific antibodies were detected in the sera of MVA-EBOV-NP and MVA-EBOV-GP-vaccinated mice. The detection of NP-specific antibodies appears slightly better compared to GP-specific antibodies, but this is most likely related to the abundance of the two viral proteins in EBOV particles that are used as an antigen in the ELISA. From day 18 to day 31, the amount of EBOV-specific antibodies increased significantly for both groups (Figure 2B). Next, the neutralizing capacity of the sera of the vaccinated mice was analyzed (Figure 2C). Priming of mice with MVA-EBOV-GP induced circulating antibodies which neutralized EBOV reaching a geometric mean titer of 1:136 at 18 dpv. The GP-specific booster immunization did not further increase the neutralization capacity of the sera (geometric mean neutralizing titer of 1:144, 31 dpv) (Figure 2C). In contrast, we observed only a slight increase in EBOV neutralization activity above background in some sera of MVA-EBOV-NP-immunized animals which was reproducible but did not reach statistical significance.

### 3.3. NP-Specific CD8+ T Cell Responses in MVA-EBOV-NP-Vaccinated Mice

To evaluate whether the MVA-EBOV-NP candidate vaccine can activate an EBOV-NP-specific CD8+ T cell response, we vaccinated IFNAR^-/-^ mice with 10^8^ PFU of recombinant MVA-EBOV-NP via the intramuscular route in a prime and prime-boost immunization scheme (Figure 2A). We tested splenocytes for EBOV NP_44–52_ (YQVNNLEEI) and NP_388–396_ (FQQTNAMVT) peptide-specific CD8+ T cells by IFN-γ ELISPOT eight days after the last immunization [45]. Primary immunizations with MVA-EBOV-NP elicited CD8+ T cells specific for both tested EBOV NP peptides with mean absolute numbers of about 52 IFN-γ spot-forming cells (SFC)/10^6^ splenocytes for the NP_388–396_ peptide and mean absolute numbers of about 89 IFN-γ SFC/10^6^ splenocytes for the NP_44–52_ peptide. The intramuscular booster immunizations markedly enhanced the T cell response to 1606 IFN-γ secreting NP_388–396_-specific CD8+ T cells/10^6^ splenocytes and 2231 IFN-γ secreting NP_44–52_-specific CD8+ T cells/10^6^ splenocytes, respectively (Figure 2D).

### 3.4. MVA-EBOV-NP and MVA-EBOV-GP-Vaccinated Mice Are Protected against Lethal EBOV Challenge

**Figure 3:**
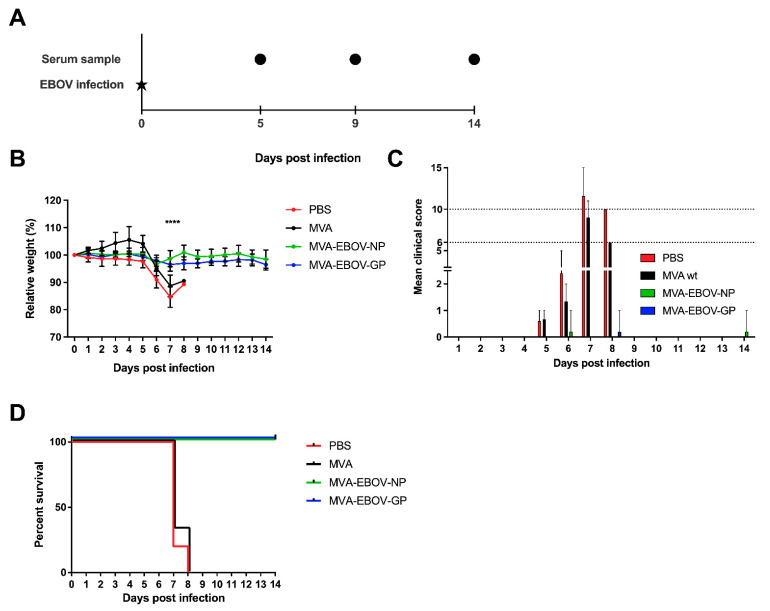
MVA-EBOV protection against EBOV infection. (**A**) Vaccinated IFNAR^-/-^ mice were challenged by intranasal inoculation with 1000 PFU EBOV (Mayinga isolate) 65 days after primary vaccination; PBS, MVA-EBOV-GP, MVA-EBOV-NP: *n* = 5 mice, MVA: *n* = 3 mice. Serum samples were obtained at the indicated time points post EBOV infection. (**B**) Mean relative body weight changes post challenge; error bars: standard deviation; **** *p* < 0.0001. (**C**) Mean clinical score comprising body weight, general condition and spontaneous behavior (see Material and Methods; Table S1). The clinical end point was defined as an individual score of 10 or of 6 on two consecutive days (dotted lines); error bars: maximum score. (**D**) Kaplan–Meier survival curves.

To determine whether the vaccine-induced immune response provided protection against EVD, vaccinated mice were challenged by an intranasal application of 1000 PFU of EBOV (Mayinga isolate; [39]) at day 65 after the first vaccination. Serum samples were collected at day 5 and 9 post infection (dpi), at the clinical end point or at the end of the study, at 14 dpi (Figure 3A). Body weight of the animals was measured daily. The infection resulted in significant weight loss in PBS- and MVA-vaccinated control mice, starting at 5 dpi. In contrast, both the MVA-EBOV-NP- as well as the MVA-EBOV-GP-vaccinated mice kept their weight until the end of the study (Figure 3B). This observation was supported by the clinical score of the animals which included body weight, general condition and spontaneous behavior which revealed no signs of clinical disease. The clinical end point was reached at an individual clinical score of ten or six on two consecutive days (Table S1). In the mock-vaccinated groups four out of five mice (PBS) or three out of four (MVA) reached the clinical end point at 7 dpi and one individual in each group at 8 dpi (Figure 3C). In contrast, the clinical score of the animals in both vaccinated groups was negligible. Only individual mice showed temporary weight loss. The survival rate in the PBS- and MVA-vaccinated control groups was 0% with mice reaching the clinical end point at days 7 or 8 pi, respectively, compared to 100% survival of both the MVA-EBOV-NP and -GP-vaccinated groups (Figure 3D; log-rank (Mantel–Cox) test *p* = 0.0002; trend *p* < 0.0001).

**Figure 4:**
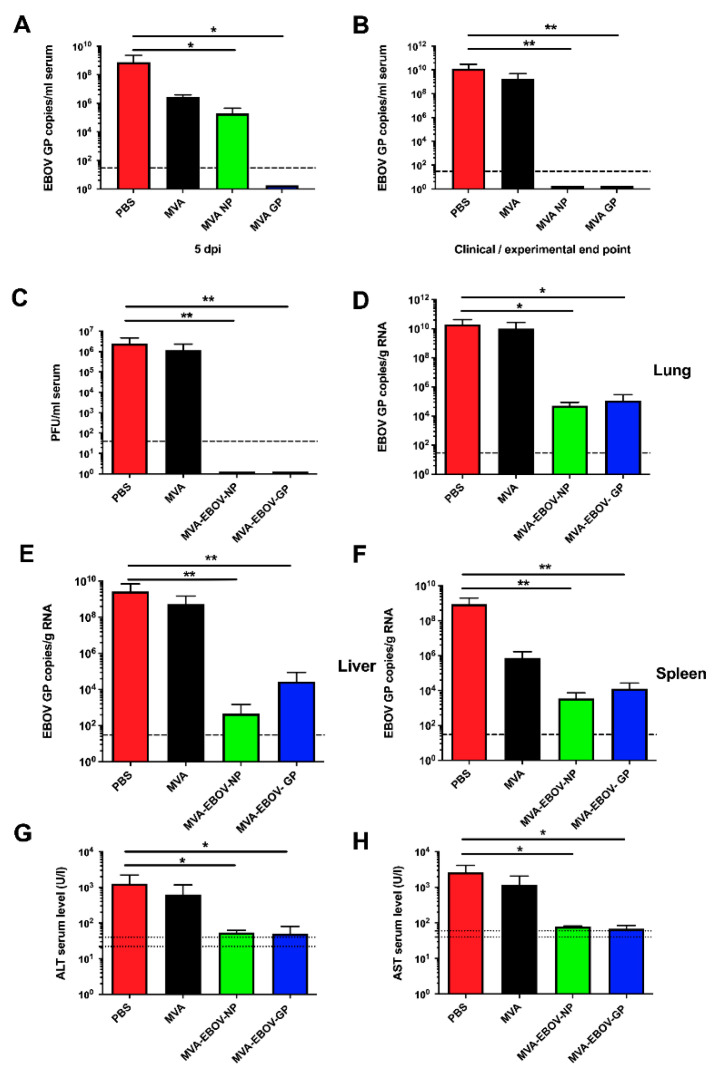
EBOV loads and clinical chemistry in infected mice. EBOV GP RNA copies in the sera at 5 dpi (days post infection) (**A**) or at the clinical (PBS, MVA: 7 or 8 dpi) or experimental (MVA-EBOV-NP, MVA-EBOV-GP: 14 dpi) end point (**B**). (**C**) Infectious EBOV in the sera at the clinical end point. EBOV GP RNA copies in lung (**D**), liver (**E**) and spleen (**F**) at the clinical end point; dashed lines: limit of detection; PBS, MVA-EBOV-GP, MVA-EBOV-NP: *n* = 5 mice, MVA: *n* = 3 mice. (**G**) ALT (alanine aminotransferase) and (**H**) AST (aspartate aminotransferase) levels at the clinical end point; dotted lines: physiological range. Error bars: standard deviation; PBS: *n* = 5, MVA: *n* = 4, MVA-EBOV-GP, MVA-EBOV-NP: *n* = 2 mice; * *p* < 0.05; ** *p* < 0.01 (one-tailed).

Virus load in sera and organs of MVA-EBOV-NP- and MVA-EBOV-GP-vaccinated mice and the control groups was assessed by quantitative real time RT-PCR (qPCR) using EBOV GP gene sequence-specific primers. Five days after the infection, sera of the PBS- and MVA-immunized mice contained more than 7 × 10^8^ and 2 × 10^6^ copies of EBOV GP RNA/mL serum, respectively. This result (involving a difference of more than two logs, Figure 4A) reveals a dramatic effect of MVA vaccination on EBOV appearance in serum, which may be transient since it is essentially gone later in infection (Figure 4B). The exact mechanism of this effect is currently enigmatic. While in MVA-EBOV-GP-vaccinated mice no EBOV RNA was detectable in the sera, MVA-EBOV-NP-vaccinated mice were viremic but did show a significant reduction of the viral load in comparison to the control groups (Figure 4A). At the experimental end point (14 dpi), all sera of MVA-EBOV-NP- and MVA-EBOV-GP-vaccinated animals were free of detectable EBOV GP-specific RNA. In contrast, the sera of PBS- and MVA-vaccinated mice still contained high EBOV loads as shown by increased levels of GP-specific RNA copies when they reached the clinical end point at 7 or 8 dpi as defined by the clinical scoring (Figure 4B). Titers of infectious EBOV in the sera of the infected mice were determined by immune plaque assay at the clinical end point (7 or 8 dpi) or at the end of the experiment (14 dpi). While infectious EBOV was neither detectable in the MVA-EBOV-NP- nor the MVA-EBOV-GP-vaccinated mice, sera of mock-vaccinated mice displayed titers of up to 10^6^ PFU/mL (Figure 4C). Additionally, we performed qPCR of homogenized lung, liver or spleen tissues. At the clinical end point we detected a high viral load in the organs of PBS-immunized mice (mean of >10^10^, 10^9^ and 10^8^ EBOV GP copies/g total RNA, respectively) and MVA-immunized mice (mean of >10^10^, 10^8^ and 10^5^ EBOV GP copies/g total RNA, respectively) (Figure 4D–F). In contrast, EBOV GP RNA was below the limit of detection in the sera of vaccinated mice at the experimental end point at 14 dpi. The organ homogenates of MVA-EBOV-NP- and MVA-EBOV-GP-vaccinated mice still contained EBOV RNA, however, at dramatically reduced copy numbers compared to the organs of mock-vaccinated animals (by a factor of approx. 10^5^, Figure 4D–F). Liver enzymes (aspartate aminotransferase (AST) and alanine aminotransferase (ALT)) were about 10-fold increased in mock-vaccinated mice (7 or 8 dpi, clinical end point) when compared to those measured in MVA-EBOV-NP or –GP vaccinated animals (14 dpi, experimental end point) (Figure 4G,H).

**Figure 5:**
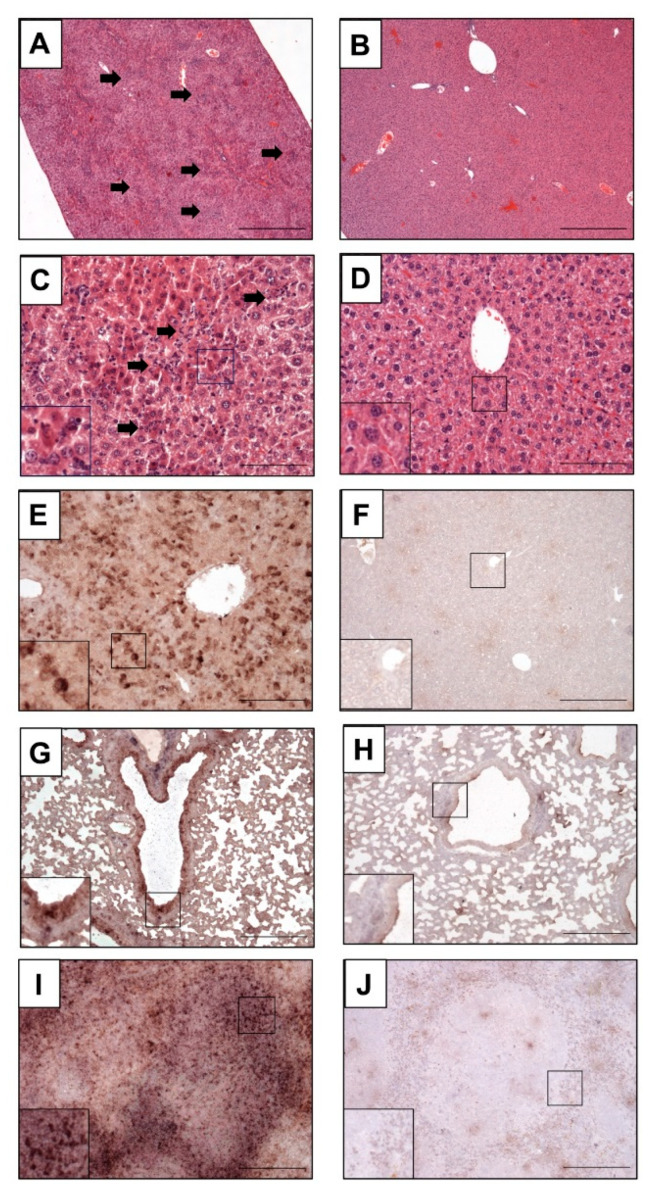
Histopathological examination of infected mice. (**A**,**C**,**E**,**G**,**I**) Mock-vaccinated control mice (PBS) compared to MVA-EBOV-NP-vaccinated mice (**B**,**D**,**F**,**H**,**J**); (**A**–**D**) H&E staining, (**G**–**J**) in situ hybridization with digoxigenin-labeled RNA probes binding to EBOV GP mRNA, brown staining; (**A**–**F**): liver, (**G**,**H**): lung, (**I**,**J**): spleen. Livers of PBS mock-vaccinated mice (**A**,**C**) showing randomly distributed hepatocellular necrosis and lymphohistiocytic infiltrations (arrows). Insert: magnification of selected areas; Scale bar (**A**,**B**): 500 µm; (**C**,**D**): 100 µm; (**E**–**J**): 200 µm.

Histomorphological analyses revealed disseminated and randomly distributed hepatocellular necrosis and lymphohistiocytic infiltrates in the PBS- or MVA-vaccinated mice, which were drastically reduced or fully absent in MVA-EBOV-NP- and MVA-EBOV-GP-vaccinated mice (Figure 5A–D). Even though not all organs showed massive histopathologic alterations in the PBS- or MVA-vaccinated mice compared to untreated, uninfected control mice (Figure S2), in situ hybridization consistently revealed the presence and the widespread organ distribution of EBOV RNA as shown exemplarily for liver, lung and spleen using EBOV GP mRNA-specific probes (Figure 5E,G,I). In contrast, and supporting the data from qPCR analyses, in organs from MVA-EBOV-NP- or MVA-EBOV-GP-vaccinated individuals the presence of EBOV GP-specific RNA was greatly reduced (Figure 5F,H,J).

### 3.5. Depletion of CD8+ T Cells in MVA-EBOV-NP-Vaccinated Mice Leads to Severe Disease Manifestation

**Figure 6:**
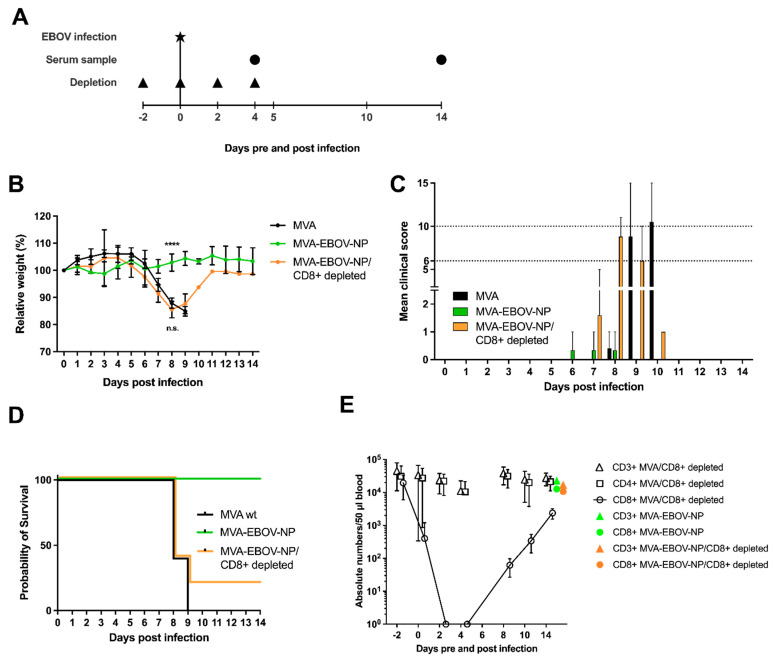
CD8+ T cells are required for protective MVA-EBOV-NP immunization. (**A**) IFNAR^-/-^ mice were vaccinated as described above with either 10^8^ PFU non-recombinant MVA (MVA) as control or with 10^8^ PFU of the MVA-EBOV-NP; MVA, MVA-EBOV-NP/CD8+ depleted: *n* = 6 mice, MVA-EBOV-NP: *n* = 3 mice. Depletion of CD8+ T cell was carried out by intraperitoneal injection of an anti-CD8+ antibody at the indicated time points. MVA-and MVA-EBOV-NP-vaccinated mice, as well as MVA-EBOV-NP-vaccinated and depleted mice were challenged with EBOV at day 65 after primary immunization. Depleted MVA-vaccinated mice were used as controls. Serum samples were obtained at the indicated time points after infection. (**B**) Mean relative body weight changes post challenge; error bars: standard deviation; **** *p* < 0.0001. (**C**) Mean clinical score comprising body weight, general condition and spontaneous behavior. The clinical end point was defined as an individual score of 10 or of 6 on two consecutive days (dotted lines); error bars: maximum score. (**D**) Kaplan–Meier survival curves. I FACS analysis for CD3+, CD4+ and CD8+ T cells. Numbers of the respective T cell population of MVA-vaccinated (CD8+ depleted) mice (*n* = 4) compared to one MVA-EBOV-NP-vaccinated (not CD8+ depleted) mouse and one surviving MVA-EBOV-NP-vaccinated (CD8+ depleted) mouse (data summarized in one graph for better visualization). 14 dpi numbers of CD8+ T cells of MVA-EBOV-NP-vaccinated (not CD8+ depleted) and surviving MVA-EBOV-NP-vaccinated (CD8+ depleted) mouse are comparable to non-infected control mice.

To better understand the immunological correlates of protection by MVA-EBOV-NP vaccination (Figure 2C), mice were vaccinated as described before with MVA-EBOV-NP and the CD8+ T cells were depleted at days −2, 0, 2 and 4 pre- and post-infection by intraperitoneal administration of a monoclonal antibody directed against CD8+ T cells [50]. MVA-EBOV-NP- and non-recombinant MVA-vaccinated but not CD8+ T cell depleted mice served as controls (Figure 6A). As seen in previous experiments, the MVA-EBOV-NP-vaccinated mice did not lose weight upon EBOV infection until the end of the trial (Figure 6B). In contrast, the CD8+ T cell depleted mice underwent significant weight loss until 8 dpi, whereby no differences between the CD8+ depleted and the non-recombinant MVA-vaccinated mice were observed (Figure 6B). However, one mouse of the MVA-EBOV-NP-vaccinated and CD8+ T cell depleted group started to gain weight again from 8 dpi onwards and resumed the initial body weight at 14 dpi. While the MVA-EBOV-NP-immunized animals only developed low clinical scores, which were mainly due to temporary weight loss, we determined comparable high clinical scores both in mice vaccinated with non-recombinant MVA or in MVA-EBOV-NP vaccinated animals upon CD8+ T cell depletion (Figure 6C). Survival rate was 100% for the MVA-EBOV-NP-vaccinated individuals and 0% for the MVA mock-immunized mice. One out of five MVA-EBOV-NP-vaccinated and CD8+ depleted mice did not reach the clinical end point until 14 dpi in spite of EBOV infection and temporary severe weight loss (Figure 6D; log-rank (Mantel-Cox) test *p* = 0.0355; trend not statistically significant). To analyze the kinetics of the antibody-mediated depletion of CD8+ T cells, we used non-infected MVA-immunized and anti-CD8+ treated mice and monitored T cells in the peripheral blood of the animals (Figure 6E). The amount of CD3+ and CD4+ T cells was determined to show specific depletion of CD8+ T cells. After two injections, corresponding to 2 dpi in the challenge experiment, no CD8+ T cells were detectable in the blood of depleted mice. However, at 8 dpi, corresponding to four days after the 4th antibody injection, we observed clearly rising numbers of CD8+ T cells coinciding with the increase of body weight seen in the surviving MVA-EBOV-NP-vaccinated and anti-CD8-depleted mouse. To investigate if rebounding CD8+ T cells could also be detected in the surviving mouse after the 4th depleting antibody injection, we measured the amounts of CD3+ and CD8+ T cells in the surviving individual and an MVA-EBOV-NP-vaccinated mouse. Indeed, we found comparable levels of CD3+ and CD8+ T cells in the peripheral blood of both animals (Figure 6E).

## 4. Discussion

The large EBOV outbreaks in West Africa and the Democratic Republic of Congo underscored the urgent need for a vaccine against EVD not only to protect individuals at risk but also to rapidly prevent spreading of the disease. During the EBOV outbreak tremendous efforts were taken to further develop previously produced vaccine candidates which had proven their efficacy in animal models [10,72]. Especially the clinical development of rVSV-ZEBOV and the combination of Ad26.ZEBOV-GP with MVA-BN-Filo was accelerated. Moreover, a phase III clinical trial in Guinea revealed efficacy of the rVSV-ZEBOV vaccine candidate [11,12,13]. However, on the one hand, the relatively frequent side-effects of rVSV-ZEBOV vaccination underline the remaining need for efficient and well tolerated vaccines suitable to routinely vaccinate medical staff in endemic areas as well as other populations at risk including children, elderly and immunocompromised individuals. On the other hand, a prime-only or a homologous prime-boost regimen could reduce the organizational and regulatory effort needed for the heterologous prime-boost regimen of the Ad26-EBOV/MVA-BN-Filo approach.

Concerning the requirements for advanced EBOV vaccine development, our MVA vaccine platform holds substantial promise because of its excellent safety profile [27,28], accompanied by potent immune stimulating properties, as a homologous prime-boost vaccine [33,34,35,36]. Indeed, recombinant MVA is highly suitable to stably express multiple EBOV gene sequences [73] and MVA vector vaccines delivering the EBOV GP antigen alone or together with the EBOV VP40 antigen have already been shown to mediate robust protection against harsh EBOV infections using different schemes of immunization in several animal models [15,37,74]. Domi et al., [37] were able to demonstrate that a single dose of an MVA-based vaccine targeting the EBOV VP40 and GP antigens was protective in a nonhuman primate model, underscoring the potency of the MVA vector platform. Previous studies in other preclinical models had suggested the contribution of EBOV NP to vaccine efficacy [43,44,46,75,76]. The efficacy of NP-specific immunization seemed to vary depending on the use of different vaccine modalities and infection models. Yet, the co-delivery of EBOV NP and GP antigens clearly enhances the protective capacity of prime-boost vaccination with DNA and adenoviral vector vaccines in non-human primates [43] and the immunization of mice with a Venezuelan equine encephalitis replicon encoding NP only can protect BALB/c mice against lethal infection with mouse-adapted EBOV [44]. However, Sullivan et al. demonstrated that application of vaccines encoding less cytotoxic GP mutants, together with NP, led to a less pronounced immune response and decreased survival in a non-human primate model [76]. In contrast, combination of wildtype GP and NP led to a protective immune response [43,76]. These findings underscore that the correlates of protection are not fully understood, and the interaction of different viral antigens needs special attention during vaccine development.

For the current study, we generated and compared two different MVA-based vaccine candidates expressing either EBOV NP (MVA-EBOV-NP) or GP (MVA-EBOV-GP). Confirming previous data, vaccination of mice with the MVA-EBOV-GP vaccine candidate resulted in a protective immune response with the development of virus-neutralizing antibodies which seem to be a major correlate of protection for this particular target protein and this EBOV vaccine. This observation is in line with previously published studies of other vaccine platforms in animals and men, also targeting EBOV GP [10,11,12,13,40]. Although MVA-EBOV-GP fully protected mice against disease and EBOV was absent in the sera of the animals, the vaccination did not prevent EBOV infection since remnant viral RNA was detected in lung, liver and spleen (Figure 4D–F).

Mice that had been vaccinated with MVA-EBOV-NP also demonstrated solid immunity against EBOV. Indeed, we did not detect any signs of body weight loss or other obvious disease signs following the infection (Figure 3B–D). To better understand the basis of this solid protective efficacy, MVA-EBOV-NP-induced immune responses were further investigated. While MVA-EBOV-NP did not induce significant levels of EBOV-neutralizing antibodies, high amounts of EBOV-binding antibodies were already detectable after the first vaccination (Figure 2B,C). However, at present, it is not possible to explain the slight neutralizing activity of sera from MVA-EBOV-NP vaccinated mice which might have been induced by Fc-receptor mediated functions, activation of the complement system or inhibition of viral release as suggested for other viruses like the influenza A virus [77]. Moreover, we were able to detect substantial cellular immune responses induced by MVA-EBOV-NP as demonstrated by high levels of IFN-γ producing CD8+ T cells for two different NP peptide specificities (Figure 2D). The booster immunization strongly increased the number of NP-specific T cells showing that homologous booster vaccination with MVA vector is able to significantly strengthen an antigen-specific CD8+ T cell response. Thus, the protection elicited by MVA-EBOV-NP vaccination is hypothesized to be exerted mainly by cytotoxic CD8+ T cells. In agreement with this hypothesis, we detected EBOV-specific RNA in the serum of MVA-EBOV-NP-vaccinated mice at 5 dpi indicating that the raised immune response did not prevent infection (Figure 4A). However, at the end of the 14 days observation time, the MVA-EBOV-NP-vaccinated mice had fully cleared the infection and neither viral RNA nor infectious virus was detectable (Figure 4B,C). The difference in the course of the infections after MVA-EBOV-GP or MVA-EBOV-NP vaccination indicated different mechanisms of the protective immune responses. The underlying mechanism of protection after MVA-EBOV-GP vaccination is probably the neutralizing antibody response which is able to prevent the majority of EBOV particles from entering the target cells. In contrast, for MVA-EBOV-NP vaccination, it is likely that the induced T cell response clears the infection only after cells are infected and T cell epitopes are presented at the plasma membrane.

Histomorphologic alterations, mainly affecting the liver in control animals, were drastically reduced or fully absent and nearly no viral RNA was detectable by in situ hybridization in lung, liver and spleen of vaccinated mice (Figure 5E,G,I). Interestingly, these findings were not substantially different with MVA-EBOV-NP-vaccinated mice, which, at 14 dpi, had cleared the infectious virus from sera suggesting that the MVA-EBOV-NP-induced viral clearance was very efficient. Importantly, depletion of CD8+ T cells in MVA-EBOV-NP vaccinated mice rendered mice susceptible to EBOV infection and abrogated protection from severe disease or death (Figure 6B–D). This data underscored the essential role of MVA-EBOV-NP induced CD8+ T cells in mediating the efficient clearance of EBOV. Our observation is supported by results from others, as an adoptive transfer of NP-specific effector T cells could protect unvaccinated mice from lethal EBOV challenge [44]. One of the NP-vaccinated and CD8+-depleted mice recovered after suffering from substantial body weight loss. This observation may be best explained by reconstitution of NP-specific CD8+ T cells after depletion. When investigating the kinetics of CD8+ T cell detection following our antibody-mediated depletion, we found that the time point of recovery in this animal coincided with the recurrence of the CD8+ T cells in the blood of antibody-treated animals (Figure 6E). Thus, it cannot be excluded that the clinical course of the EBOV infection in this particular animal was slightly delayed compared to the other mice with the consequence that the reappearance of CD8+ T cells was timely to control the severe disease. The recurrence of CD8+ T cells might be due to activation of CD8+ T cell memory, stimulated by repeated antigen exposure [78]. Overall, our data clearly suggest the beneficial effect of MVA-induced NP-specific T cell immunity for protection against EBOV. Similarly, cytotoxic CD8+ T cells have been found essential for rapidly protective MVA vaccination against lethal mousepox virus infection [50,51]. Indeed, there might be a more general requirement of pathogen-specific CD8+ T cells for rapidly protective immunizations, and NP-specific MVA immunization could be a useful component of EBOV emergency immunization. Based on the results in the present study, we will investigate the effect of combining NP with other target antigens, such as GP and VP40, to possibly obtain synergistic effects regarding a protective immune response to EBOV-specific vaccination using recombinant MVA or other vaccine modalities.

In summary, both analyzed MVA-based anti-EBOV vaccine candidates protected IFNAR^-/-^ mice against EBOV challenge infection. Vaccination with MVA-EBOV-GP resulted in the production of virus-neutralizing antibodies and vaccination with MVA-EBOV-NP induced NP-specific cytotoxic CD8+ T cells. Both neutralizing antibodies and NP-specific CD8+ T cells were equally able to generate a protective immune response and are therefore promising candidates for further vaccine development. The current results highlight the potency of MVA-based vaccines to elicit a strong and protective CD8+ T cell response. Given the susceptibility of neutralizing antibody responses to antigen mutations, as witnessed during the SARS-CoV-2 pandemic, this important feature of the MVA vector urgently needs further investigation.

## Data Availability

The data that support the findings of this study are available from the corresponding author upon reasonable request.

## Supplementary Materials

The following supporting information can be downloaded at: https://www.mdpi.com/article/10.3390/vaccines10040533/s1, Table S1: Clinical scoring of EBOV-infected mice, Figure S1: Plasmid maps of pIIIH5red-EBOV-GP and pLW-73-EBOV-NP, Figure S2: Tissue of non-infected control mice.
