# Supplementary material for: Protective CD8+ T Cell Response Induced by Modified Vaccinia Virus Ankara Delivering Ebola Virus Nucleoprotein
Source: Vaccines (Basel). 2022 Mar 29;10(4):533. doi: 10.3390/vaccines10040533 (PMC9027530; doi:10.3390/vaccines10040533)
Supplement: Supplementary file 1 [file vaccines-10-00533-s001.zip › vaccines-1632622-supplementary.pdf]

Supplement Table S1: Clinical scoring of EBOV-infected mice.

**Score**	**0**	**1**	**5**	**10**
**Body weight**	0-5 % difference to mean individual body weight*	6-10 % difference to mean individual body weight*	11-15 % difference to mean individual body weight*	>15 % difference to mean individual body weight*
**General condition**	Fur smooth, shiny; body orifices clean, eyes clean and shiny	Fur slightly ruffled, limited or exaggerated coat care	Dull fur; limited care of body orifices; eyes dull	Fur sticky or shaggy and ruffled; diarrhea; icterus or signs of coagulopathy
**Spontaneous behavior**	Normal behavior (sleeping, reaction to touch, curiosity, social contacts)	Slight change of normal behavior	Moderate changes of activity (hyper-/ hypoactivity)	Massive changes of activity (hyper-/ hypoactivity); lethargy, isolation, self-aggression or aggressive behavior against cage mates

* Measured on 3-5 days prior to infection.

**Total score**	**Degree of severity**	**Explanation**
0	None	No physical burden, daily control (incl. weight)
1-3	Low	Low burden, daily control (incl. weight) and additional daily adspectorial control
5-7	Moderate	Moderate burden; clinical end point is reached if score is 6 or higher on two consecutive days
10 or higher	High	Heavy burden; clinical end point is reached if score is 10 or higher

Supplement Figure S1: Plasmid maps of pIIIH5red-EBOV-GP and pLW-73-EBOV-NP.

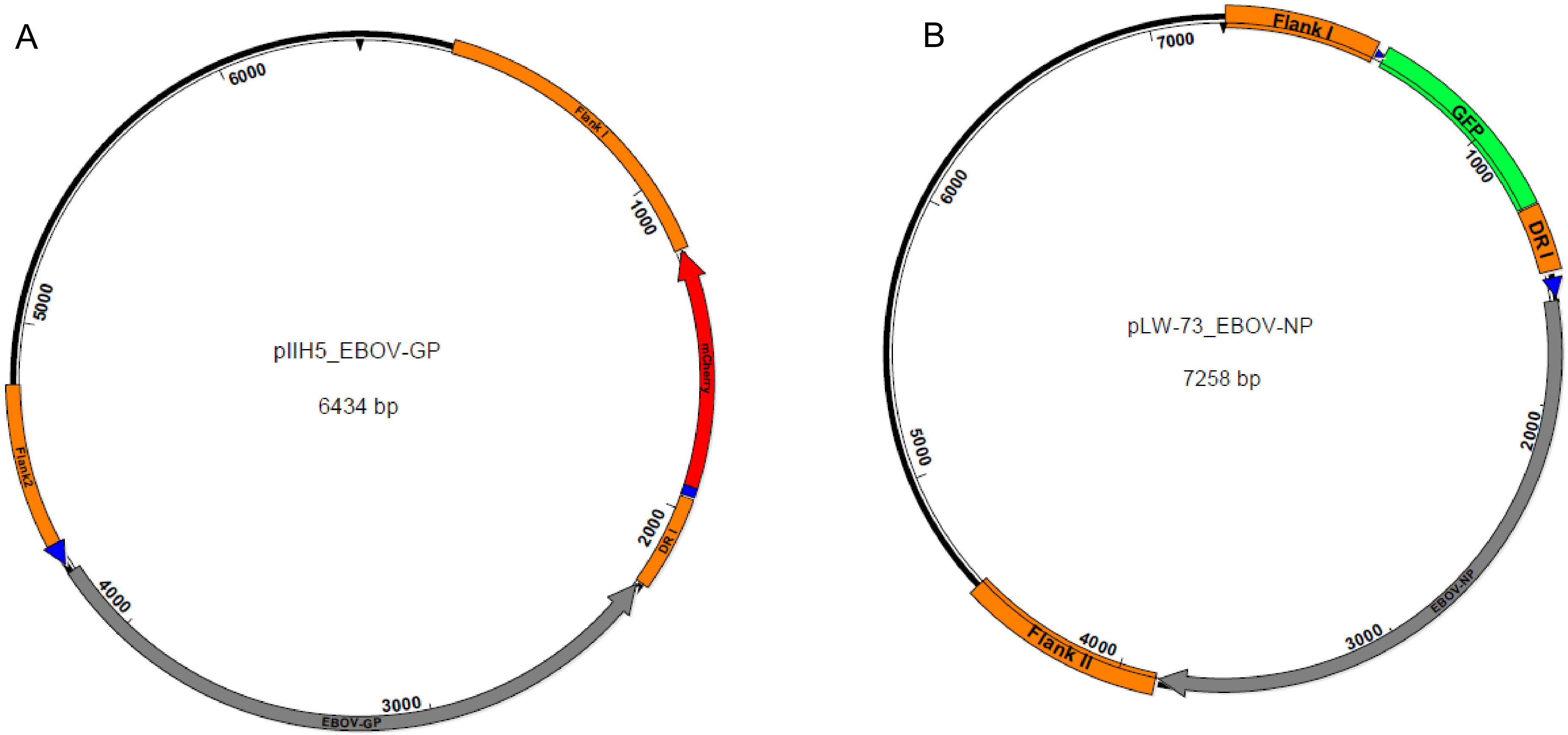

Figure S1: Plasmid maps of pIIIH5red-EBOV-GP (A) and pLW-73-EBOV-NP (B). Flank-1 and flank-2 refer to MVA DNA sequences targeting the deletion site III (A) or the intergenic site 069R-070L (B) in the MVA genome for insertion of recombinant genes encoding for EBOV glycoprotein (EBOV-GP) or for EBOV nucleoprotein (EBOV-NP), Short repetitive sequences of MVA DNA (DR I) served to remove the marker genes (mCherry, GFP) by intragenomic homologous recombination (marker gene deletion).

Supplement Figure S2: Tissue of non-infected control mice.

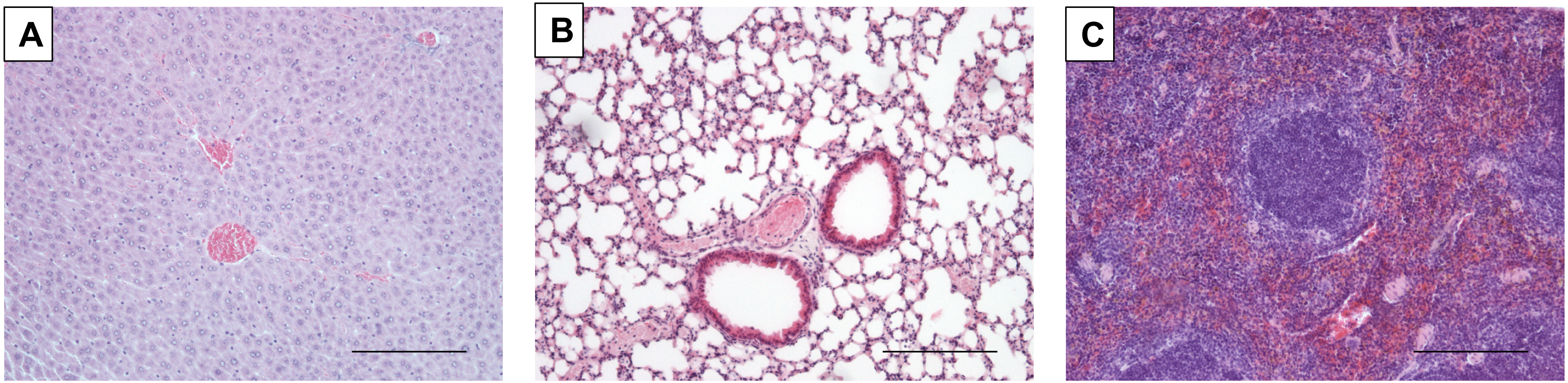

Figure S2: Tissue of non-infected control mice. (A) liver, (B) lung, (C) spleen of untreated IFNAR$^{-/-}$ mice; H&E staining. Scale bar: 200 µm.
